# Fullerenol increases effectiveness of foliar iron fertilization in iron-deficient cucumber

**DOI:** 10.1371/journal.pone.0232765

**Published:** 2020-05-04

**Authors:** Nikolai P. Bityutskii, Kirill L. Yakkonen, Kseniia A. Lukina, Konstantin N. Semenov

**Affiliations:** 1 Department of Agricultural Chemistry, Saint Petersburg State University, Saint Petersburg, Russia; 2 Department of General and Bioorganic Chemistry, First Pavlov State Medical University, Saint Petersburg, Russia; Huazhong Agriculture University, CHINA

## Abstract

The water-soluble fullerenols are novel carbon-based nanomaterials with unique properties, which afford them with wide agricultural applications. Iron (Fe) deficiency is the most common and widespread nutrition disorder affecting plants. Foliar Fe treatments of plants have been carried out with solutions devoid of fullerenol. In this study, the role of fullerenol [C_60_(OH)_22–24_] in alleviation of Fe deficiency in *Cucumis sativus* (a Strategy I plant) via foliar fertilization was investigated. Cucumber plants were grown hydroponically, either with (Fe) or in Fe-free (−Fe) nutrient solution. The following foliar spray treatments were applied: fullerenol at final concentrations of 1 (F1) and 10 (F10) mg L^-1^; Fe(II)SO_4_·7H_2_O; Fe(II)-EDTA (ethylenediaminetetraacetic acid); and Fe(II)-F1 and Fe(II)-F10. The best used compound was a combination of Fe(II)-sulfate with fullerenol, especially Fe-F1. The addition of fullerenol to Fe(II)-sulfate solutions significantly increased leaf-active Fe (extracted by an Fe(II) chelator) and re-greening at the site of application. The fullerenol-induced mutual influences did not appear when fullerenol was sprayed alone, suggesting a beneficial role of Fe(II)–fullerenol interactions in the penetration of Fe(II) in the leaves and re-greening under Fe-limited conditions. The results are of importance to enhancing the potential of foliar Fe fertilization as the commonly used strategy for ameliorating Fe deficiency and improving crop yield and quality.

## Introduction

Iron (Fe) plays numerous functions in key metabolic processes, and its essentiality for higher plants was discovered in the middle of the 19^th^ century [1, 2]. Despite being most abundant in the earth’s crust, a deficiency of Fe caused by its low solubility in soils at moderate and high pH (alkaline and calcareous soils) is a common nutritional disorder affecting crops in many areas of the world [3, 4]. Leaf chlorosis (yellow leaf colour) is a typical symptom of Fe deficiency; additionally, Fe deficiency impairs crop quality and yield [5]. To acquire Fe under Fe-limited conditions, plants evolved adaptive mechanisms. The so-called Strategy I plants (dicotyledonous and monocotyledonous species, except grasses) respond to an Fe deficit by: (i) enhanced H^+^-ATP-ase mediated proton extrusion by roots; (ii) induction of a plasma membrane ferric chelate reductase (FC-R) enzyme; and (iii) increased Fe^II^ transport [1, 6, 7]. Strategy II plants (grasses) release phytosiderophores (PSs) to chelate Fe^III^ in the rhizosphere and form soluble Fe^III^-PS complexes, which are imported by specific plasmalemma transporter proteins [8]. However, under natural Fe chlorosis conditions, Fe uptake in plants, especially in Strategy I plants, may be significantly impaired, for example, by the presence of bicarbonate ion (HCO_3_^-^) in calcareous soils, which may neutralize root proton (H^+^) release and block the expression of H^+^-ATPase (*HA1*), ferric reductase (*FRO*), and Fe transporter (*IRT1*) genes in Fe-deprived roots [9, 10].

The problem of Fe limitation can be overcome by using Fe fertilizers. While the supply of Fe-chelates is the most efficient practice to control Fe chlorosis in crops, foliar sprays which facilitate the delivering of small amounts of Fe to plants can be a cheaper and more environmentally-friendly strategy to address Fe deficiency in crops [11, 12]. In the oriental plane tree, foliar Fe-containing sprays improved biological functions of plants even more effectively when compared with soil-applied treatments [13]. Moreover, foliar Fe fertilization is an effective way to promote Fe concentrations and bioavailability of polished rice [14]. Rapid progress has been made in evolution of foliar iron fertilization and elucidating pathways for Fe uptake in leaves [11, 12, 15–17]. In these works, different Fe compounds, surfactants and other adjuvants have been studied to increase the effectiveness of foliar Fe applications.

With respect to optimizing Fe spray formulation, engineered nanomaterials (ENMs) could have great potential due to their small surface area and enhanced reactivity compared with equivalent bulk materials [18–20]. Among these, carbon-based nanomaterials have increasing environmental and agricultural applications in recent years [21]. Fullerenes are an allotropic modification of carbon with unique structural and electronic properties that enable numerous application fields, including agricultural applications [21–24]. Fullerene C_60_ and its water-soluble derivates, such as fullerenol or fullerol (F), are the best-investigated fullerenes. Whilst the majority of studies report negative or no effects of fullerene C_60_ on plants, fullerenol, as OH-functionalized fullerene, induces positive effects on plant growth [21]. It has been reported that fullerenol increased biomass, fruit yield and phytomedicine content in bitter melon (*Momordica charantiai*) [25]. Fullerenol treatment enhanced root elongation of barley (*Hordeum vulgare*) under stressful conditions, such as UV-B radiation, salt stress and an excess of salicylic acid [26]. At certain concentrations of fullerenol, seedlings of *Arabidopsis thaliana* exhibited longer hypocotyls [27]. Foliar application of fullerenol nanoparticles alleviated oxidative effects of drought stress in sugar beets [28]. It has been hypothesized that fullerenol can act as a scavenger of free radicals under stress conditions [26] or as an intracellular binder of water, enabling adaptation of plants to drought impact [28]. Although the exact mechanisms of fullerenol-induced mutual influences are not yet clear, these findings demonstrated perspectives of fullerenol for agricultural applications. Information on the relevance of fullerenol in foliar Fe fertilization of plants suffering Fe deficiency is still lacking.

For this reasons, the objective of this study was to determine whether fullerenol is of importance for foliar Fe fertilization of Strategy I model plants (*Cucumis sativus* L.) under Fe-limiting conditions.

## Materials and methods

### Fullerenol synthesis and identification

**Fig 1:**
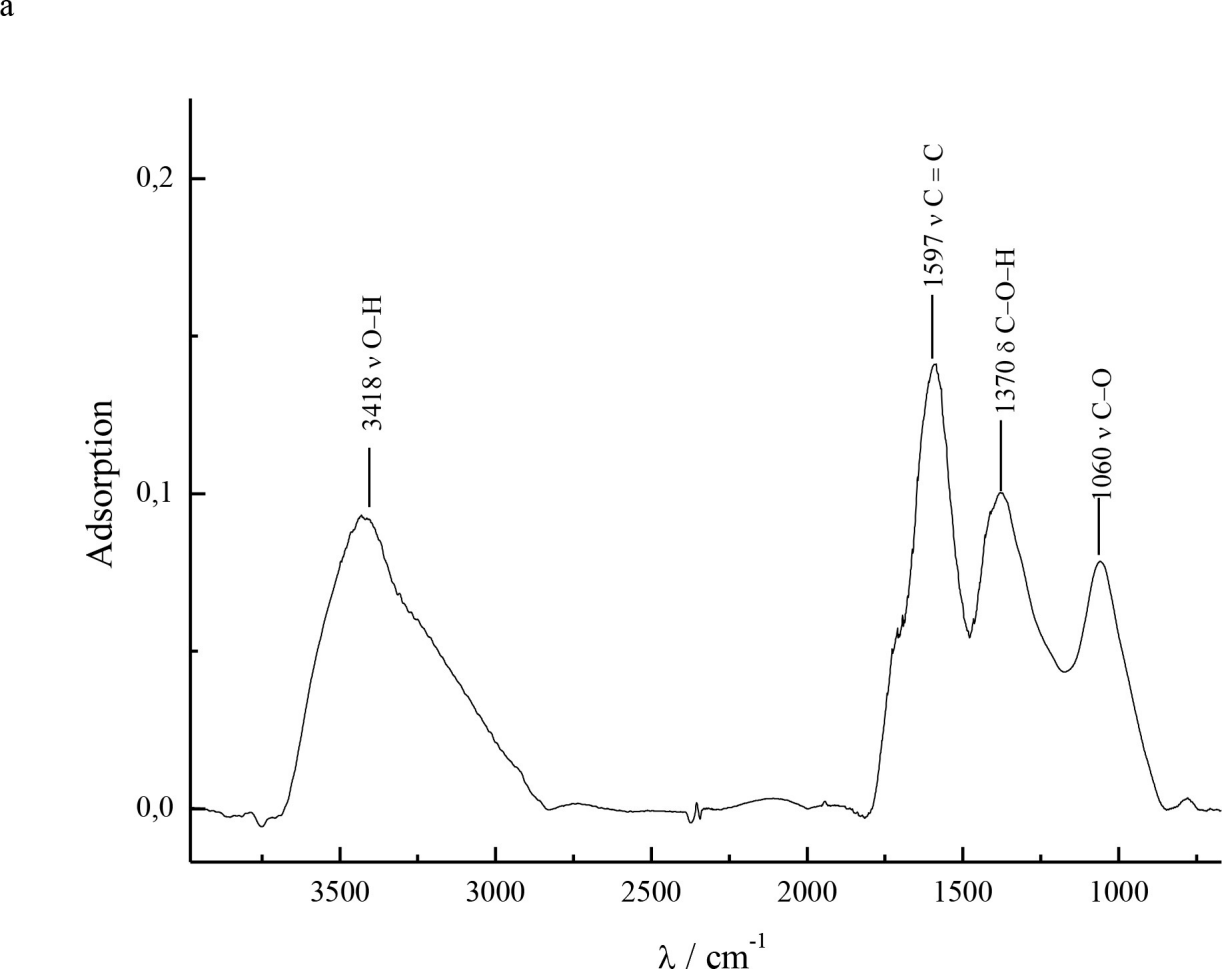
IR spectrum of C_60_(OH)_22–24_ sample.

Fullerenol (C_60_(OH)_22-24_) was synthesized, as described recently [29, 30]. A group of physicochemical methods was used in order to identify the fullerenol: IR spectroscopy (Shimadzu FTIR-8400S spectrometer, Japan), elemental analysis (EuroVector Euro EA3028-HT, Italy), mass spectrometry (Shimadzu MALDI-TOF mass spectrometer Axima–Resonance, Japan), UV spectroscopy (Shimadzu UV-1800 spectrophotometer, Japan), _13_C NMR spectroscopy (NMR spectrometer Bruker Avance III 400 WB, USA), complex thermal analysis (Shimadzu DTG-60H). FTIR: 3418 cm^–1^ (νO–H), 1597 cm^-1^ (νC = C), 1370 cm^-1^ (δ_S_C–O–H) and 1060 cm^-1^ (νC–O) (Fig 1). Experimental elemental analysis data: (C: 63.72%; H: 2.22%), calc.: (C: 63.83%; H: 2.13%). According to the elemental analysis results, a relative molar weight of 1128 g mol^–1^ was considered in all further calculations (corresponds to C_60_(OH)_24_).

The negative ion MALDI-TOF mass spectrum of fullerenol indicates ion fragmentation, at the same time presence of a molecular ion at *m*/*z* equal to 1094–1128 reveals that fullerenol consists of 22–24 hydroxyl groups. The UV/Vis spectrum of the fullerenol aqueous solution does not contain the absorption peak at 335 nm typical for individual C_60_ fullerene (S1 Fig). Fullerenol UV/Vis spectra are not informative enough, however they can be applied for the composition determination (as an example, in the wavelength range 300–500 nm, in which the absorption is not too high).

**Fig 2:**
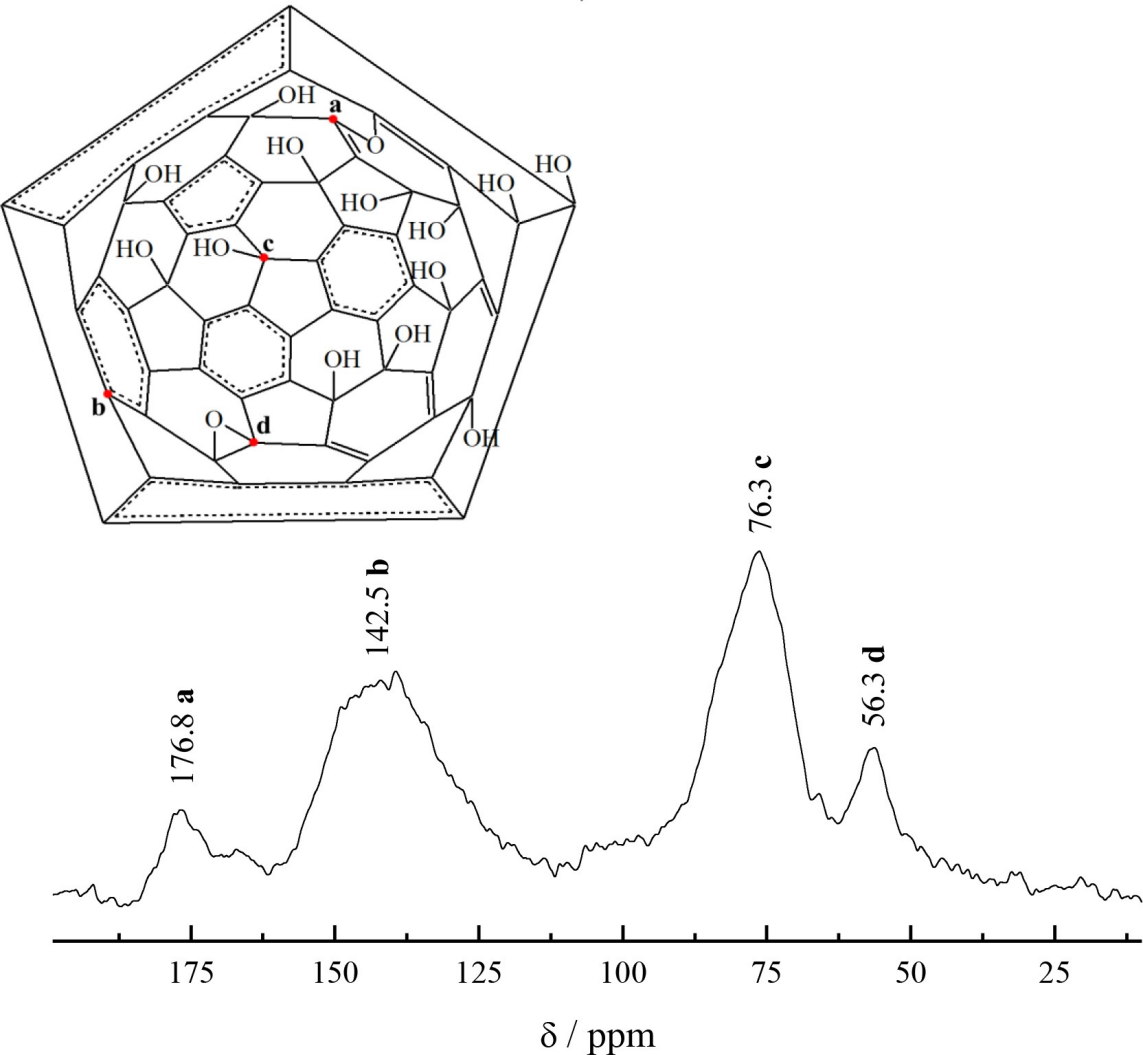
^13^С NMR spectrum of fullerenol obtained using CP/MAS technique at contact time 2 ms.

The ^13^C NMR spectrum obtained using the CP/MAS technique is shown in Fig 2. An analysis of this spectrum indicates: (i) a peak in the region of 176.8 ppm can be referred to the carbon atoms in the fullerene molecule bonded through the inbuilt oxygen atom (Fig 2A); (ii) a peak at 142.5 ppm corresponds to the structurally “pure” carbon nuclei in the stoichiometric fullerene molecule (Fig 2B), (iii) a peak at 76.3 ppm corresponds to hydroxylated carbon atoms (Fig 2C); (iv) a peak at 56.3 ppm can be identified as carbon atoms of the epoxy group (Fig 2D).

The NMR spectra of C_60_(OH)_22–24_ obtained by exciting a signal with different pulse sequences are shown in S2 Fig. The above-mentioned spectral lines can be distinguished in both presented spectra. At the same time, in the case of direct excitation (DE) technique, the lines in the region of 60 ppm and 175 ppm have low intensity, which indicates that the studied modified fullerene has no improperly modified elements.

The results of complex thermal analysis demonstrate that (i) the C_60_(OH)_22–24_ fullerenol is thermally stable up to *T* = 340 K; (ii) the fullerenol decomposition starts in the temperature interval *T* = 340–1100 K and may consist of processes of dehydroxylation, hemiketal formation and pinacol type rearrangement of hydroxyl groups and their degradation (S3 Fig). The mass loss in the temperature interval *T* = 298–1100 K equal to 36.17% is related to the loss of 22–24 hydroxyl groups; the process of the fullerene core decomposition starts at *T* = 1100 K. Thus, the thermal analysis can be applied for the identification of fullerenols.

### The hydrodynamic diameters and ζ-potentials of fullerenol associates

Table 1 presents the hydrodynamic diameters and ζ-potentials of associates in binary (C_60_(OH)_22–24_–H_2_O) and ternary (C_60_(OH)_22-24_−FeSO_4_–H_2_O) systems. The results demonstrate that even at low concentrations, aqueous fullerenol solutions were associated. The addition of FeSO_4_ used for foliar fertilization (pH 4.0) led to a significant increase in the degree of association and particle size. Values of *ζ*-potential were negative, indicating that all solutions were electrokinetically stable.

**Table 1:** Hydrodynamic diameters and ζ-potentials of associates in binary (C_60_(OH)_22–24_ –H_2_O) and ternary (C_60_(OH)_22-24_–FeSO_4_–H_2_O) systems at pH 4.0.

Sample	Hydrodynamic diameter (nm)	ζ-potential (mV)
1 mg L^-1^ C_60_(OH)_22-24_	21	**–**30
10 mg L^-1^ C_60_(OH)_22-24_	140	**–**39
2 mM FeSO_4_ + 1 mg L^-1^ C_60_(OH)_22-24_	613	**–**40
2 mM FeSO_4_ + 10 mg L^-1^ C_60_(OH)_22-24_	621	**–**40

### Plant material and growth conditions

Cucumber seeds (*Cucumis sativus* L., cv. Phoenix) were obtained from VIR plant genetic resources (Russia). Cucumber seeds were surface-sterilized and germinated between two sheets of filter paper, moistened with distilled water for four days in the dark at 28ºC. Then, the seedlings were pre-incubated for 7 days in a complete nutrient solution containing (mM): 1.0 KCl, 3.0 Ca(NO_3_)_2_, 0.5 MgSO_4_, 1.0 KH_2_PO_4_, and (μM): 1.0 MnSO_4_, 1.0 ZnSO_4_, 0.5 CuSO_4_, 0.01 (NH_4_)_6_Mo_7_O_24_ and 10 H_3_BO_3_. Iron (Fe) was supplied as Fe^III^-EDTA at 10 μM. The pH of nutrient solutions was adjusted to 6.0. After 7 days of pre-culture, the plants were transferred to 1-L plastic pots (three plants per pot) and exposed to the same nutrition solution, either with (+Fe) or in Fe-free (−Fe) nutrient solution. The following foliar spray treatments were applied: 1) distilled water; 2) fullerenol at final concentrations of 1 (F1) mg L^-1^; 3) fullerenol at final concentrations of 10 (F10) mg L^-1^; 4) Fe(II)SO_4_·7H_2_O; 5) Fe(II)-EDTA; 6) Fe(II)-F1; and 7) Fe(II)-F10. All Fe treatments contained Fe at final concentrations of 2 mM. The Fe-EDTA was freshly prepared by complexing Fe(II) (FeSO_4_·7H_2_O) with EDTA (ethylendiaminetetraacetic acid) at 1:1 (Fe:ligand); and Fe-F1 and Fe-F10 were freshly prepared by mixing Fe(II) (FeSO_4_·7H_2_O) with F1 and F10. To keep Fe soluble and to retard the process of oxidation, FeSO_4_·7H_2_O formulations were kept at pH 4.0 [15]. For comparability, all other sprays were also adjusted to pH 4.0. Foliar spraying was performed once using a hand sprayer after 5 days of Fe deficiency when Fe-deficient symptoms became distinctly apparent. A volume of 2 ml of the treatment solutions was sprayed per pot approximately 4 h after the onset of the illumination. To mimic typical field spraying techniques, both leaf sides were treated until full wetting. To avoid unknown interactions with fullerenol, surface-active agents and other adjuvants were not yet tested in this study. The nutrient solutions were completely renewed every 2–3 days and continuously aerated. Plants were grown at 24 ± 2ºC: 20 ± 2ºC (light: dark) with a day/night regime of 16/8 h and a photon flux density of 200 μmol m^-2^ s^-1^ at plant height.

### Chlorophyll (Chl) determination and growth analysis

The chlorophyll content in leaves from three different positions (from the base to the youngest leaf: L1, L2 and L3) was monitored non-destructively for 7 days after treatments as spectral plant analysis diagnostic (SPAD) units, using a portable Chlorophyll Meter SPAD-502 device (Minolta Camera Co., Osaka, Japan). Averaged SPAD measurements from four different locations were taken per leaf, and three leaves at each position were monitored per pot (12 different leaves per treatment). Whereas leaf 1 (L1) developed during Fe treatment (pre-incubation period for 7 days with Fe), the other leaves (L2 and L3) appeared after the pre-incubation period. At harvest, the pigments were extracted from the middle part of the fully expanded second leaves 2 (L2), using pure acetone, and the absorbance was measured at 662 (Chl *a*) and 644 (Chl *b*) nm, as described elsewhere [31]. Final Chl concentrations were expressed on a dry leaf weight basis. At harvest, the plants were divided into the following parts: root; stem (together with leaf petioles); and leaves (blades), and they were subsequently dried at 70ºC and weighed.

### Determination of ferric chelate reductase (FC-R) activity in roots

The ferric chelate reductase (FC-R) activity was determined at the end of experiments by measuring the formation of the Fe(II)-BPDS [Fe(II)-bathophenanthroline-disulfonic acid disodium salt hydrate] complex from Fe(III)-EDTA [Fe(III)-ethylenediaminetetraacetic acid], as described by Pavlovic and colleagues [32]. The pH of the assay solution was adjusted to 5.5. After 0.5 h of incubation in darkness at 25ºC, absorbance at 535 nm was determined with a spectrophotometer (model Speks SSP-705, Spektroskopicheskie sistemy, Russia).

### Determination of active Fe of leaves

Prior to determination, leaves were washed, first with 0.1% Tween 80 acidulated with 0.1 M HCl, and then with distilled water [33]. The so-called “active iron” was determined by the method developed by Abadía and colleagues [34], using 2.2´-bipyridyl. The reagent (83 mM) was in water, and the pH was 3.0 (HCl). At the end of experiments, the leaves (L2, 0.5 g) were incubated with the reagent for 24 h, and the absorbance was measured at 520 nm.

### Statistical analysis

Data were subjected to analysis of variance procedures (ANOVA, type III), using IBM SPSS Statistics (version 21), and mean values were compared by the Student–Newman–Keuls post-hoc test at a 5% significance level (*P* < 0.05). Four replicate pots were used per treatment. Pearson’s coefficient (*r*) was determined to test whether the investigated parameters were correlated.

## Results

### Leaf Chl and plant growth

**Fig 3:**
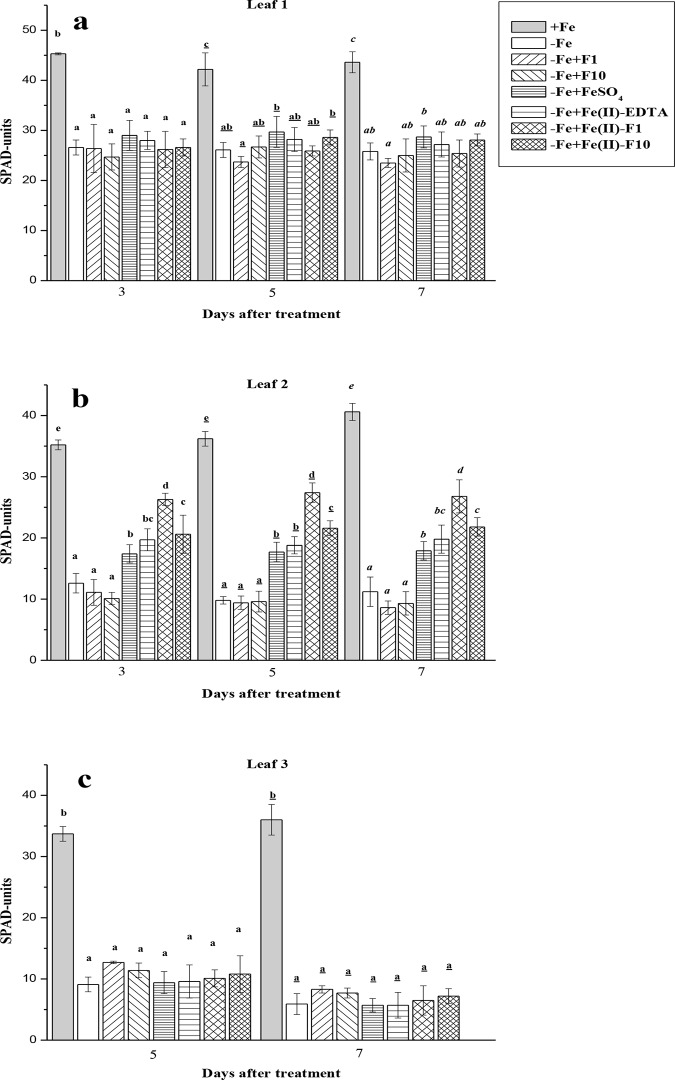
SPAD units in cucumber leaves at different positions grown hydroponically in a nutrient solution. Data are expressed as mean ± standard deviation (n = 4). Significant differences between treatments (*P* < 0.05) are indicated by different letters.

**Fig 4:**
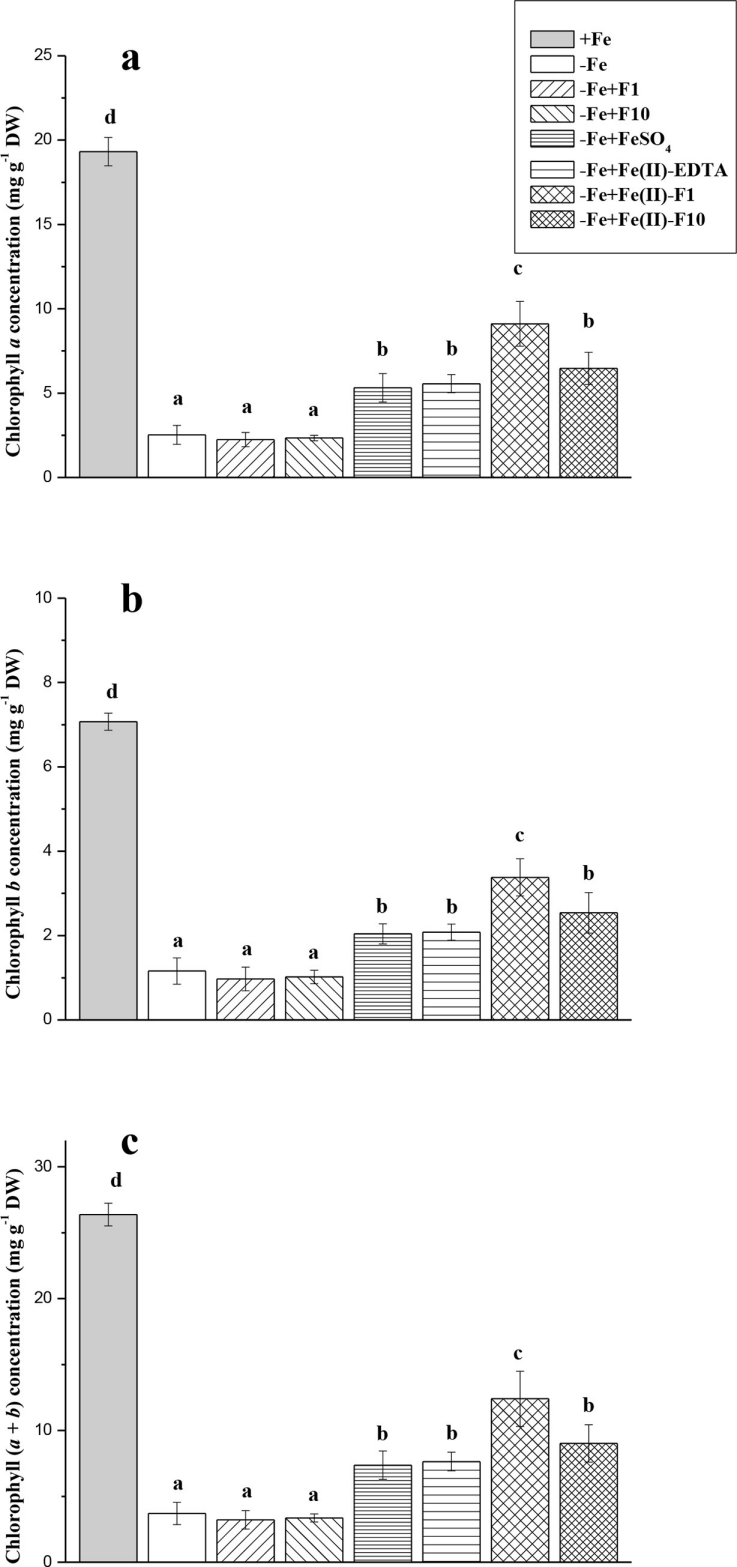
Concentrations of chlorophyll *a*, chlorophyll *b* and chlorophyll *a + b* in the second leaves of cucumber grown hydroponically in a nutrient solution. Data are expressed as mean ± standard deviation (n = 4). Significant differences between treatments (*P* < 0.05) are indicated by different letters.

Cucumber plants grown without Fe showed severe chlorosis symptoms (Figs 3 and 4). In −Fe plants, for example, the SPAD units of leaves at different positions were 1.7–6.0-fold lower compared with +Fe plants, with the highest expression of the differences in leaves (L2 and L3) already having formed after the end of the +Fe-pre-incubation period (Fig 3). At harvest, the Fe-limiting conditions decreased the concentrations of both Chl *a* and Chl *b* in the second leaves, on average, by 7-fold in comparison with Fe-fed plants (Fig 4). A single foliar spray with different Fe compounds induced distinct re-greening effects, which were more pronounced in the second leaves treated with Fe-F1 at the end of the experiments (Figs 3 and 4). Whereas Fe(II)-sulfate increased SPAD values and Chl (*a* + *b*) by 1.6- and 2.0-fold, respectively, Fe-F1 treatments increased these indices by 2.4- and 3.3-fold, respectively (Figs 3 and 4). Generally, the positive effects of Fe(II)-sulfate and Fe(II)-EDTA on leaf Chl and SPAD were identical. In contrast, in–Fe leaves sprayed with Fe-free fullerenol solutions, the re-greening effects were not obtained throughout the experiments (Figs 3 and 4). The SPAD units of the non-sprayed third leaves were not affected with foliar treatments, including even treatments with different Fe compounds (Fig 3C).

**Fig 5:**
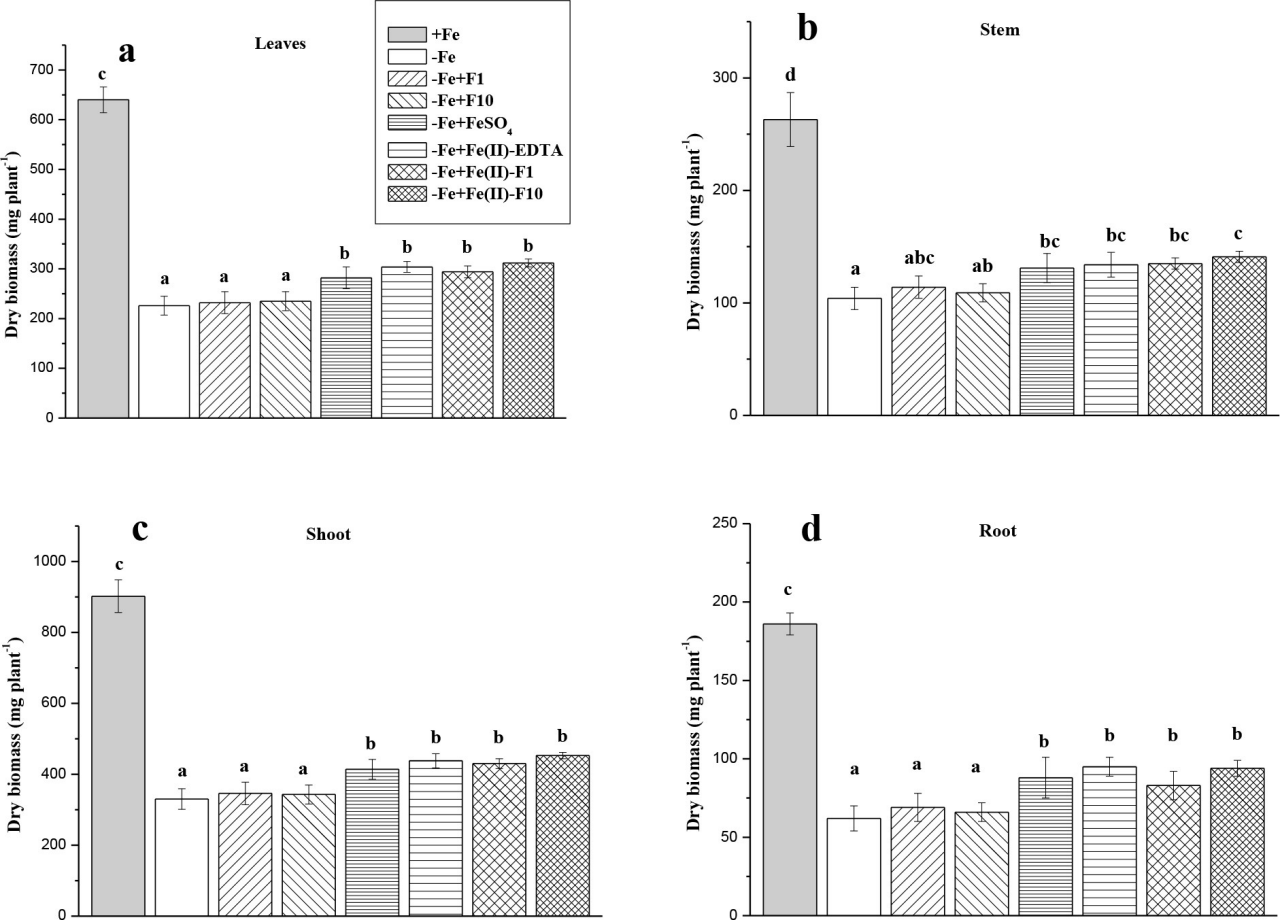
Biometric data in cucumber grown hydroponically in a nutrient solution. Data are expressed as mean ± standard deviation (n = 4). Significant differences between treatments (*P* < 0.05) are indicated by different letters.

The chlorosis symptoms induced by Fe deficiency (Figs 3 and 4) were accompanied by a significant decrease in the dry biomass of cucumber leaves (−65%), stem (−60%) and roots (−67%) (Fig 5). With all Fe-containing compounds, the dry biomass of cucumber plants increased significantly (on average by 30%), irrespective of Fe-compounds. Fullerenol treatments without Fe did not significantly affect plant growth suffering Fe deficiency (Fig 5).

### Leaf-active Fe and FC-R activity of roots

**Fig 6:**
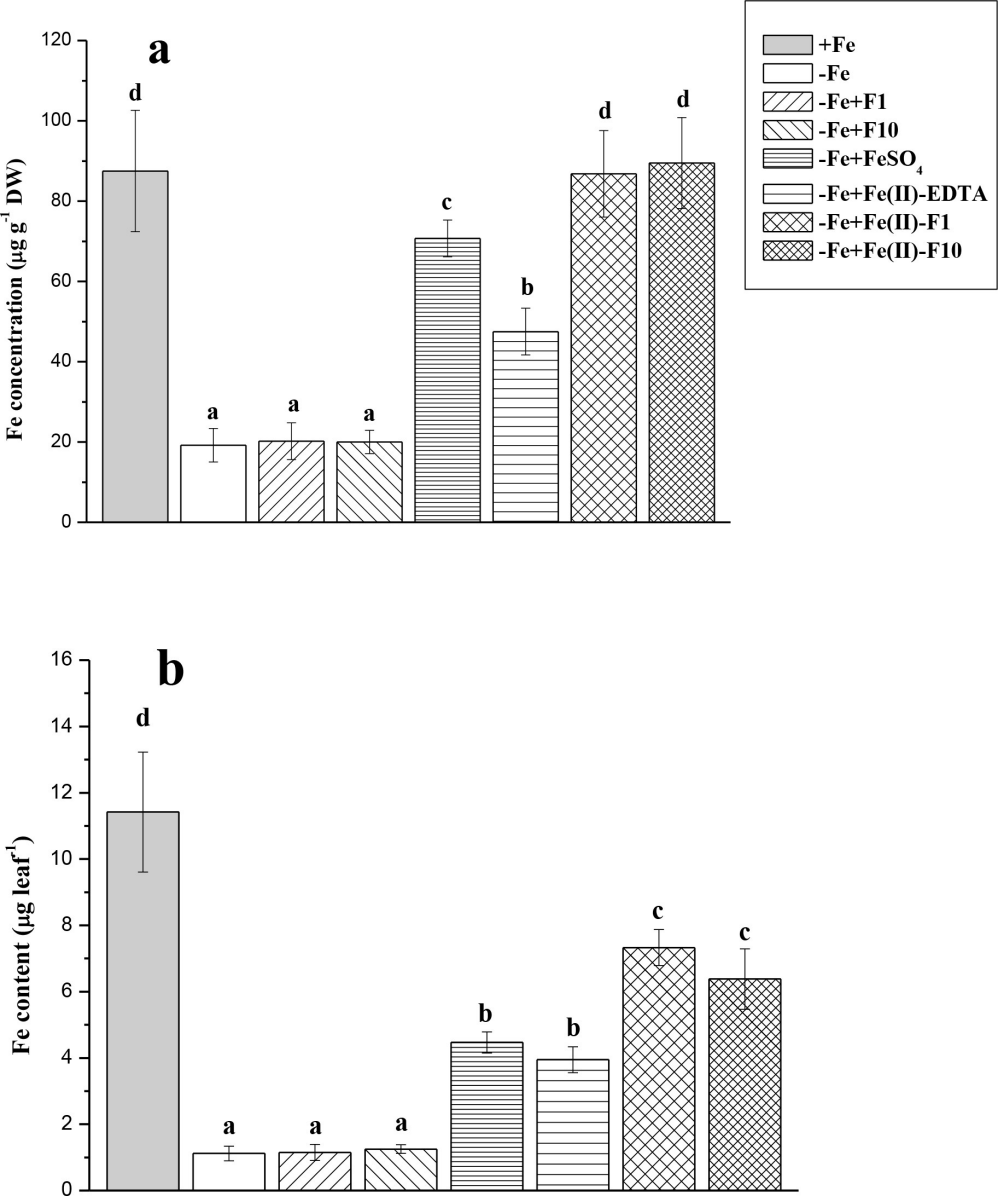
Active Fe concentration and content in the second leaves (L2) of cucumber grown hydroponically in a nutrient solution. Data are expressed as mean ± standard deviation (n = 4). Significant differences between treatments (*P* < 0.05) are indicated by different letters.

At the end of the experiment, −Fe plants exhibited significantly decreased amounts of active Fe extracted by Fe(II) chelator in cucumber leaves compared with Fe-sufficient plants (Fig 6). In leaves treated with Fe compounds, the concentration and content of active Fe was significantly higher than in non-Fe-treated plants (Fig 6). Using Fe with fullerenol (F1 or F10) led to the best results in terms of increases of leaf Fe concentration/content (Fig 6). The chelate Fe(II)-EDTA was no more effective than Fe(II)-sulfate when active Fe content was investigated (Fig 6B). A high correlation between leaf-active Fe concentration and Chl (*a* + *b*) content was found (*r* = 0.958, *P* = 0.01) (Figs 4 and 6).

**Fig 7:**
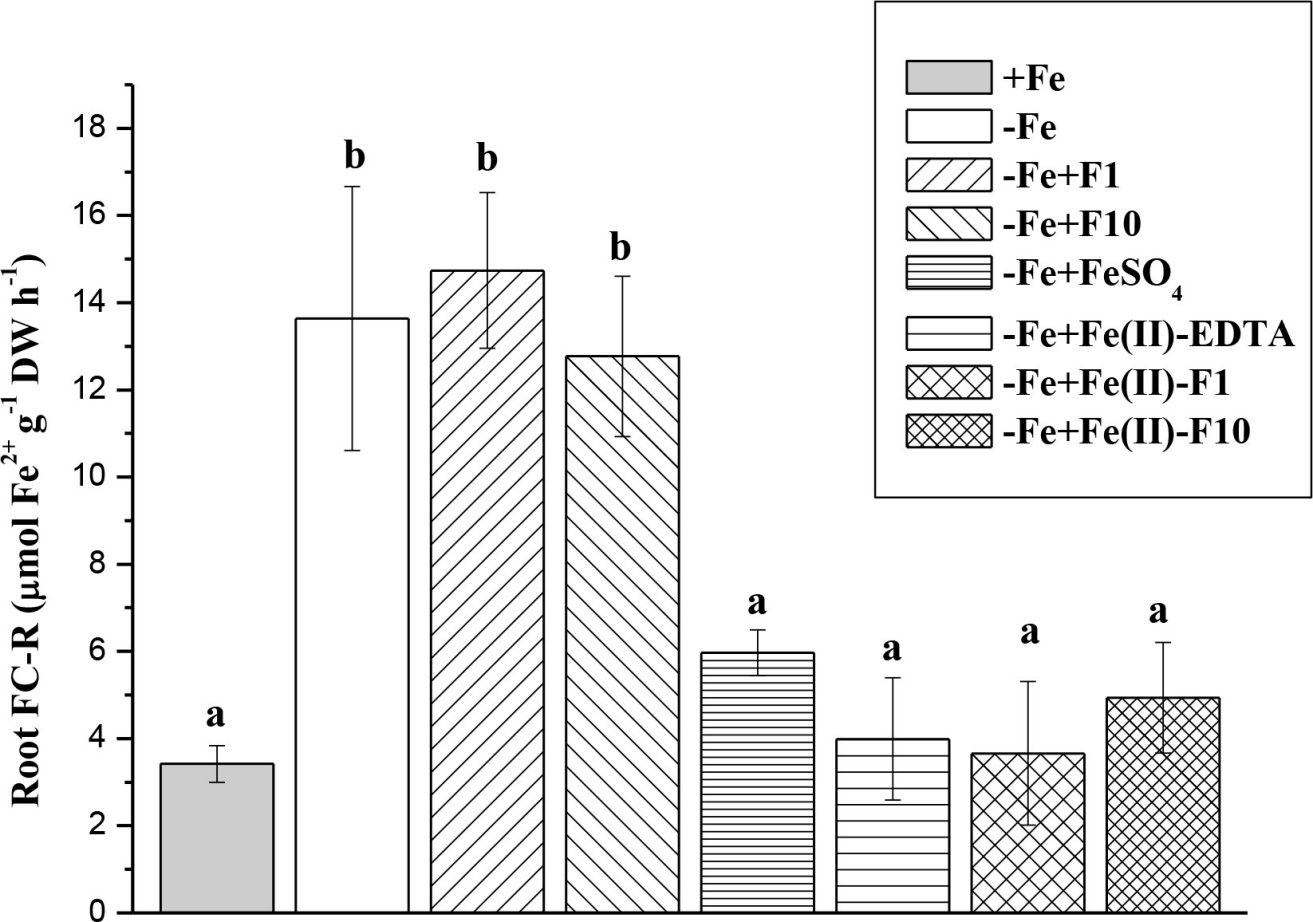
Root ferric chelate reductase (FC-R) activity of cucumber grown hydroponically in a nutrient solution. Data are expressed as mean ± standard deviation (n = 4). Significant differences between treatments (*P* < 0.05) are indicated by different letters.

At the end of the experiment, the cucumber roots responded to a lack of Fe in a nutrient solution by increasing FC-R by 4 times (Fig 7). Foliar Fe applications decreased the FC-R to the level of Fe-fed plants, irrespective of Fe-containing compounds. The leaf treatment with fullerenol alone (without Fe) did not significantly alter the root FC-R of Fe-stressed plants.

## Discussion

### The effects of foliar Fe fertilization

Leaf yellowing (chlorosis) is the most evident symptom of Fe deficiency in higher plants [1]. In this study, the Fe-starved cucumber plants expressed severe symptoms of Fe chlorosis: a significant decrease in leaf Chl (tested either non-destructively (SPAD units) or destructively); and a significant decrease in active Fe (Figs 3, 4 and 6). The decrease in Chl concentration induced by Fe deficiency was accompanied with a decrease in plant biomass (Fig 5) and an increase in the FC-R of roots (Fig 7). Reduction (through secretion of compounds with reducing properties or expression of an FC-R) is an important mechanism affecting Fe bioavailability in the rhizosphere [35]. In Strategy I plants, ferrous Fe is a substrate for trans-membrane transport [36].

Single foliar Fe treatments of Fe-deficient cucumber were generally biologically effective. Indeed, the Fe-treated plants exhibited a significant increase in leaf Chl (re-greening L2), leaf-active Fe (L2) and dry biomass compared with the non-treated plants grown under Fe-limiting conditions (Figs 3–6). Moreover, in the treated plants, the root FC-R decreased to the level of the Fe-sufficient plants, indicating that Fe supply of the Fe-treated plants became better when they were grown in the absence of Fe in a nutrient solution (Fig 7). However, foliar Fe spray differentially affected the leaf re-greening depending on Fe-containing formulations. In this study, the best compound was a combination of Fe(II)-sulfate with fullerenol, especially with F1 (Fe-F1), when considering the final Chl increase (Figs 3 and 4). The highest amounts of extractable (active) Fe in cucumber leaves were also observed when fullerenol was added in Fe-containing spray formulations (Fig 6), suggesting that fullerenol increased the cellular labile pool of Fe(II), which is responsible for the greening of chlorotic plants [34, 37]. The transitory pool of cell Fe metabolism is defined as a pool of redox-active Fe associated with different ligands [38]. At the same time, no significant re-greening was obtained in the absence of Fe in the acid spray solutions with fullerenol, irrespective of its concentration (F1 or F2) (Figs 3–7). Although foliar acid treatments alone (without Fe) may be quite effective in re-greening Fe-deficient leaves [39], the effect is obvious when significant amounts of pre-exiting Fe pools occur in Fe-chlorotic leaves [40]. Taken together, our results suggest that fullerenol can promote the leaf penetration of Fe(II)-sulfate applied to the foliage, and thereby re-greening Fe-starved cucumber. The treatments, however, did not lead to leaf Chl and active Fe as high as those found in Fe-sufficient cucumber plants.

### The mechanisms involved in the Fe uptake in leaves

The mechanisms involved in the Fe uptake in leaves are still poorly known [15]. Ionic species are thought to cross leaf cuticles through aqueous pores which are very small in size [41]. Also, stomatal pathways can be an alternative route to uptake foliar sprays [42]. There was no correlation between molecular mass and penetration rates for different Fe compounds. Moreover, the cuticular membrane permeability decreased with the concentration of Fe-chelates, suggesting that Fe-chelates may reduce the size of aqueous pores [43]. Furthermore, it has been reported that Fe(II)-sulfate applied as an acidic solution was the best Fe-compound in foliar fertilization experiments carried out with pear and peach trees [11, 40]. Applied to the foliage, Fe of Fe(II)-sulfate entered most in palisade and spongy parenchyma of leaves [12]. The mechanisms of Fe(II) entrance in the treated area of the leaf is still poorly known. On the one hand, Fe(II) may directly enter the leaf cell via Fe(II) transporters [40]. On the other hand, Fe(II) may be oxidized and then chelated with apoplastic ligands, such as organic acids and nicotianamine. In the latter case, the foliar effectiveness of Fe(II)-sulfate would be determined by the functioning of FC-R in leaf plasma membranes [11]. Physico-chemical characteristics of Fe sprays are also the factors determining the effectiveness of foliar Fe fertilization [11].

### The possible role of fullerenol in foliar Fe fertilization

How fullerenol acts to improve foliar Fe(II) fertilization of cucumber still remains unclear. It seems plausible that the mechanism is not directly connected with the ability of fullerenol to improve solubility of applied Fe. In this study, foliar treatment with a highly soluble Fe(II)-chelate (Fe-EDTA) was not more biologically effective than treatment with Fe(II)-sulfate at the same pH values (Figs 3 and 4). Therefore, solubility of Fe(II) was not a limiting factor for Fe uptake in cucumber leaves, at least when acidic spray solutions (pH 4.0) were applied. Moreover, several authors have concluded that Fe(II)-sulfate is better in alleviating Fe-chlorosis than synthetic Fe-chelates via foliar fertilization [11, 40]. Possibly, fullerenol may improve chemical stability of applied Fe(II), which is of importance for preventing oxidation of Fe(II) to Fe(III), and therefore, for direct uptake of Fe(II) through a transporter. Many authors agree that the beneficial effects of fullerenol nanoparticles on plants are due to their antioxidant activity–that is, an ability to serve as a scavenger of free radicals [26, 28]. Recently, it has been reported that interactions between carbon nanotubes and the Fe ions may have a role in the reduction of Fe(III) to Fe(II) oxidation state [44]. Very recently, it has been demonstrated that fullerenol directly reduced Fe(III) to Fe(II) via electron transfer of fullerenol-Fe(III) complex [45]. Moreover, visible light significantly enhanced the generation of Fe(II) induced by fullerenol probably due to its high photosensitivity. It would be expected that foliar Fe fertilization with fullerenol will be more effective in light than in darkness. Furthermore, fullerenol might directly facilitate membrane transport of ferrous Fe in leaves. It has been shown that nanoparticles of fullerenol are mobile in plant tissues and can penetrate through different biomembranes [25, 28]. Fullerene C_60_ can be transported to the shoots from roots of rice plants [46]. Positive Fe(II) ions can make bonds with polyanionic nanoparticles of fullerenol, shifting its surface charge to the more positive values, and thereby creating a delivery system for Fe(II) [47]. Finally, during foliar application, fullerenol can bind large amounts of water, creating an additional water reserve which plays a vital role under drought stress [28]. Water released from fullerenol during foliar Fe fertilization would be required for a significant cuticular penetration of Fe(II)-sulfate and would favour foliar uptake.

Limited information is available on how fullerenol associates pass through the cell wall and across the plasma membrane. Nevertheless, it was clearly demonstrated that small size and good solubility allow fullerenol [C_60_ (OH)_20_] readily permeate through the cell wall of plants (*Allium cepa*) likely due to a concentration gradient [48]. Fullerenol can chelate with Fe species due to its numerous surface groups [49]. Before Fe can be absorbed it must be released from the Fe-complex [16]. Complexes Fe(II)-fullerenol are far less stable than synthetic chelates commonly used for foliar fertilization, therefore this may be no problem for the splitting of the complex by ligand exchange and further transport of Fe across the membrane. Fullerenol is hydrophilic and largely excluded by the plasma membrane. Therefore, the fullerenol was found to accumulate between the cell wall and the plasma membrane of plant cells [48]. At high concentrations (more than 30-70mg L^-1^), fullerenol can initiate cell damage, resulting in a loss of membrane integrity and fullerenol appearance within the cytoplasm. The membrane damage could affect membrane transport of nutrients [48]. However, foliar application of fullerenol even at very high concentration (e.g. 700 μmol L^-1^) did not significantly alter growth of sugar beets [28]. Nevertheless, further studies are necessary to elucidate the mechanisms involved in fullerenol-mediated Fe uptake when correction of chlorosis is carried out by foliar Fe fertilization.

Although fullerenol (especially F1) in combination with Fe(II)-sulfate was effective in increasing the Chl of treated leaves (L2), it did not alter Chl in non-treated cucumber leaves (L3) (Figs 1 and 2). The results indicate that Fe(II)-F as well as other Fe-compounds (Fe(II)-sulfate, Fe(II)-EDTA) were effective in re-greening only treated leaf areas–that is, at the site of application. El-Jendoubi and colleagues [12] also reported that the effects of the foliar fertilization of Fe-deficient peach trees and sugar beets by Fe-sulfate were minor outside the leaf surface treated. Collectively, these findings suggest that Fe has a limited re-translocation rate from uptake sites to other parts of Fe-deficient plants. Therefore, repeated foliar Fe treatments are necessary to improve Fe fertilization, even through Fe sprays contain effective adjuvants, such as fullerenol.

The cost of the complex of Fe(II)-sulfate with fullerenol is mainly determined by the cost of fullerenol. On the one hand, water-soluble derivatives of fullerenes, for example polyhydroxylated fullerenes, are currently quite expensive. According to the Sigma-Aldrich catalogue, the cost of 100 mg of fullerenol is about €500. On the other hand, the original one-stage technology of the production of polyhydroxylated fullerenes developed in our scientific group allows reducing the cost of the final product by hundreds and even thousands of times [50]. Moreover, a positive effect of this mixture was observed without adding of different surfactant and other adjuvants which widely used in foliar fertilization, with the increasing the complexity and cost of potential application. According to our preliminary estimates, the average cost per hectare of land for mixed fullerenols obtained from fullerene soot would not be in excess of €6.5

## Conclusions

The presented results demonstrated, for the first time, a prominent role of fullerenol (a carbon-based nanomaterial) in foliar Fe fertilization of Fe-deficient cucumber plants. The addition of fullerenol to Fe(II)-sulfate sprays promoted the leaf penetration of Fe, in turn increasing leaf-active (extractable) Fe and successful suppression of plant Fe-deficiency symptoms at the site of application. The re-greening effects induced by fullerenol were expressed when it was applied in combination with Fe(II)-sulfate, but not alone, suggesting that the Fe(II)–fullerenol interactions are significant for the effectiveness of Fe treatments applied to the foliage. The results are of importance to enhancing the potential of foliar Fe fertilization as the commonly used environmentally-friendly strategy to alleviate Fe deficiency in crops.

## Supporting information

S1 Fig UV/Vis spectra of C_60_(OH)_22–24_ sample with the concentration C = 0.08 g dm^–3^ (solid line) and pristine fullerene C_60_ (solution in 1,2-dimethylbenzene) with the concentration C = 0.029 g dm^–3^ (dashed line). The wavelength corresponding to the peak is λ = 335.7 nm. A—optical density.
(DOC)

S2 Fig ^13^С NMR spectrum of fullerenol obtained using DE (solid line) and CP/MAS (dashed line) at contact time 2 ms. (DOC)

S3 Fig Complex thermal analysis of C_60_(OH)_22–24_ sample in the temperature region *T* = 300–1200 K. Solid line corresponds to the *TG* curve, whereas the dotted one relates to the *DTG* curve. The inset graph is *DSC*.
(DOC)

S1 Appendix The data underlying the findings. (DOC)
