# Supplementary material for: Fullerenol increases effectiveness of foliar iron fertilization in iron-deficient cucumber
Source: PLoS One. 2020 May 4;15(5):e0232765. doi: 10.1371/journal.pone.0232765 (PMC7197802; doi:10.1371/journal.pone.0232765)
Supplement: S1 Fig — The wavelength corresponding to the peak is λ = 335.7 nm. A—optical density. (DOC) [file pone.0232765.s001.doc]

**S1 Fig. UV/Vis spectra of C_60_(OH)_22–24_ sample with the concentration C = 0.08 g dm^–3^ (solid line) and pristine fullerene C_60_ (solution in 1,2-dimethylbenzene) with the concentration C = 0.029 g dm^–3^ (dashed line).** The wavelength corresponding to the peak is λ = 335.7 nm. A — optical density.
