# Supplementary material for: Fullerenol increases effectiveness of foliar iron fertilization in iron-deficient cucumber
Source: PLoS One. 2020 May 4;15(5):e0232765. doi: 10.1371/journal.pone.0232765 (PMC7197802; doi:10.1371/journal.pone.0232765)
Supplement: S2 Fig — (DOC) [file pone.0232765.s002.doc]

# S2 Fig. ^13^С NMR spectrum of fullerenol obtained using DE (solid line) and CP/MAS (dashed line) at contact time 2 ms.
