# Supplementary material for: Fullerenol increases effectiveness of foliar iron fertilization in iron-deficient cucumber
Source: PLoS One. 2020 May 4;15(5):e0232765. doi: 10.1371/journal.pone.0232765 (PMC7197802; doi:10.1371/journal.pone.0232765)
Supplement: S3 Fig — Solid line corresponds to the TG curve, whereas the dotted one relates to the DTG curve. The inset graph is DSC. (DOC) [file pone.0232765.s003.doc]

**S3 Fig. Complex thermal analysis of C_60_(OH)_22–24_ sample in the temperature region *T* = 300–1200 K**. Solid line corresponds to the *TG* curve, whereas the dotted one relates to the *DTG* curve. The inset graph is *DSC*.
