# Supplementary material for: Fullerenol increases effectiveness of foliar iron fertilization in iron-deficient cucumber
Source: PLoS One. 2020 May 4;15(5):e0232765. doi: 10.1371/journal.pone.0232765 (PMC7197802; doi:10.1371/journal.pone.0232765)
Supplement: S1 Appendix — (DOC) [file pone.0232765.s004.doc]

**Table 1 Appendix. Hydrodynamic diameters and ζ-potentials of associates in binary (C_60_(OH)_22–24_ –H_2_O) and ternary (C_60_(OH)_22-24_–FeSO_4_–H_2_O) systems at pH 4.0**

| Sample | Hydrodynamic diameter (nm) | | | ζ-potential (mV) | | |
| --- | --- | --- | --- | --- | --- | --- |
|  | data points | median | variance measures | data points | median | variance measures |
| 1 mg L^-1^ C_60_(OH)_22-24_ | 20, 20, 22 | 20 | 1 | -30, -30, -30 | -30 | 0 |
| 10 mg L^-1^ C_60_(OH)_22-24_ | 140, 139, 140 | 140 | 0 | -40, -38, -39 | -39 | 1 |
| 2 mM FeSO_4_ +  1 mg L^-1^ C_60_(OH)_22-24_ | 615, 611, 614 | 614 | 4 | -40, -40, -40 | -40 | 0 |
| 2 mM FeSO_4_ +  10 mg L^-1^ C_60_(OH)_22-24_ | 620, 625, 618 | 620 | 13 | -40, -39.5, -39 | -40 | 0 |

**Table 2 Appendix.** SPAD units in cucumber leaves at different positions grown hydroponically in a nutrient solution

| Treatments | Leaf 1 | | | | | | | | |
| --- | --- | --- | --- | --- | --- | --- | --- | --- | --- |
|  | 3d | | | 5d | | | 7d | | |
|  | data points | median | variance measures | data points | median | variance measures | data points | median | variance measures |
| +Fe | 45.5  45.6  45.2  45.1 | 45.3 | 0.1 | 39.2  43.8  46.1  39.7 | 41.8 | 11.1 | 44.5  46.0  41.8  41.8 | 43.2 | 4.4 |
| −Fe | 27.6  26.9  27.4  24.3 | 27.2 | 2.3 | 24.6  25.1  27.7  26.9 | 26.0 | 2.2 | 25.5  27.6  26.5  23.6 | 26.0 | 3.0 |
| −Fe + F1 | | 33.5  25.5  24.3  22.5 | | --- | | 24.9 | 23.5 | 25.3  23.1  22.9  23.6 | 23.4 | 1.2 | 22.1  24.1  23.8  24.1 | 24.0 | 0.9 |
| −Fe + F10 | 26.0  27.8  23.5  21.8 | 24.7 | 7.0 | 27.1  28.7  27.5  23.7 | 27.3 | 4.7 | 25.2  20.4  28.1  26.3 | 25.8 | 10.7 |
| −Fe + FeSO_4_ | 27.1  27.0  33.4  28.8 | 27.9 | 9.0 | 26.9  30.6  33.7  27.6 | 29.1 | 9.6 | 27.4  27.9  31.9  27.7 | 27.8 | 4.6 |
| −Fe + Fe(II)-EDTA | 27.1  27.9  26.7  30.6 | 27.5 | 3.1 | 26.4  26.6  28.4  31.6 | 27.5 | 5.7 | 28.7  29.8  25.3  24.8 | 27.0 | 6.1 |
| −Fe + Fe(II)-F1 | 28.3  20.9  27.1  28.7 | 27.7 | 13.3 | 27.1  24.9  25.3  26.4 | 25.8 | 1.0 | 28.3  22.3  24.2  26.8 | 25.5 | 7.2 |
| −Fe + Fe(II)-F10 | 24.2  27.5  26.8  28.0 | 27.2 | 3.0 | 28.25  29.0  26.8  30.5 | 28.6 | 2.3 | 27.1  27.8  29.8  27.8 | 27.8 | 1.4 |
|  | Leaf 2 | | | | | | | | |
|  | 3d | | | 5d | | | 7d | | |
| +Fe | 34.3  34.6  35.6  36.1 | 35.1 | 0.7 | 34.9  37.0  37.5  35.3 | 36.1 | 1.5 | 40.5  39.6  42.7  40.0 | 40.2 | 1.9 |
| −Fe | 14.4  13.1  12.1  10.6 | 12.6 | 2.6 | 9.5  10.4  10.5  9.1 | 9.9 | 0.4 | 11.6  13.7  11.7  8.0 | 11.6 | 5.5 |
| −Fe + F1 | 11.2  10.2  14.0  9.1 | 10.7 | 4.5 | 10.9  9.0  8.2  9.8 | 9.4 | 1.3 | 9.4  8.7  7.1  9.2 | 9.0 | 1.1 |
| −Fe + F10 | 10.2  11.0  10.5  8.7 | 10.3 | 1.0 | 12.2  8.4  9.0  8.9 | 8.9 | 3.0 | 7.6  8.4  11.9  9.4 | 8.9 | 3.5 |
| −Fe + FeSO_4_ | 18.9  18.5  15.6  16.6 | 17.5 | 2.4 | 17.5  19.7  18.2  15.7 | 17.8 | 2.8 | 18.1  16.3  17.6  19.8 | 17.8 | 2.1 |
| −Fe + Fe(II)-EDTA | 22.1  17.9  19.8  19.0 | 19.4 | 3.1 | 19.2  17.1  18.6  20.6 | 18.9 | 2.1 | 22.7  18.2  17.8  20.4 | 19.3 | 5.1 |
| −Fe + Fe(II)-F1 | 25.1  27.6  26.4  26.1 | 26.2 | 1.1 | 26.8  29.1  28.4  25.6 | 27.6 | 2.5 | 23.1  29.4  26.5  28.2 | 27.4 | 7.4 |
| −Fe + Fe(II)-F10 | 17.9  20.0  19.6  25.2 | 19.8 | 9.9 | 22.3  19.8  22.2  22.2 | 22.2 | 1.4 | 21.6  24.0  20.5  21.2 | 21.4 | 2.4 |
|  | Leaf 3 | | | | | | | | |
|  | 5d | | | 7d | | |  |  |  |
| +Fe | 32.8  32.8  34.3  35.1 | 33.5 | 1.4 | 35.8  38.4  37.2  32.6 | 36.5 | 6.1 |  |  |  |
| −Fe | 8.9  10.5  10.2  8.0 | 9.5 | 1.4 | 8.4  5.8  4.9  4.8 | 5.3 | 2.8 |  |  |  |
| −Fe + F1 | 12.6  12.9  13.0  12.5 | 12.8 | 0.05 | 9.1  8.2  8.1  7.8 | 8.1 | 0.3 |  |  |  |
| −Fe + F10 | 13.2  11.7  11.0  10.3 | 11.3 | 1.5 | 7.5  7.4  8.9  6.9 | 7.4 | 0.7 |  |  |  |
| −Fe + FeSO_4_ | 9.5  8.6  7.7  11.9 | 9.1 | 3.4 | 6.9  5.1  4.5  6.3 | 5.7 | 1.2 |  |  |  |
| −Fe + Fe(II)-EDTA | 7.5  10.0  13.4  7.7 | 8.8 | 7.4 | 5.3  5.2  8.7  3.7 | 5.3 | 4.5 |  |  |  |
| −Fe + Fe(II)-F1 | 9.0  11.0  8.8  11.7 | 10.0 | 2.0 | 7.0  5.7  9.7  3.9 | 6.3 | 6.0 |  |  |  |
| −Fe + Fe(II)-F10 | 9.4  11.2  7.9  14.8 | 10.3 | 9.0 | 6.0  8.3  8.1  6.4 | 7.2 | 1.3 |  |  |  |

**Table 3 Appendix. Concentrations of chlorophyll *a*, chlorophyll *b* and chlorophyll *a + b* in** the second leaves of cucumber grown hydroponically in a nutrient solution

| Treatments | Chlorophyll *a* concentration (mg g^-1^ DW) | | | Chlorophyll *b* concentration  (mg g^-1^ DW) | | | Chlorophyll (a + *b*) concentration (mg g^-1^ DW) | | |
| --- | --- | --- | --- | --- | --- | --- | --- | --- | --- |
|  | data points | median | variance measures | data points | median | variance measures | data points | median | variance measures |
| +Fe | 20.29  18.67  19.10  19.20 | 19.15 | 0.48 | 7.30  6.83  7.14  7.01 | 7.08 | 0.04 | 27.59  25.50  26.24  26.21 | 26.23 | 0.76 |
| −Fe | 2.97  2.21  3.04  1.91 | 2.59 | 0.31 | 1.54  0.87  1.30  0.95 | 1.13 | 0.10 | 4.51  3.08  4.34  2.86 | 3.71 | 0.72 |
| −Fe + F1 | 2.24  2.38  1.69  2.70 | 2.31 | 0.18 | 1.02  1.08  0.56  1.22 | 1.05 | 0.08 | 3.26  3.46  2.25  3.91 | 3.36 | 0.49 |
| −Fe + F10 | 2.50  2.20  2.22  2.46 | 2.34 | 0.02 | 1.15  0.79  1.04  1.11 | 1.08 | 0.03 | 3.65  2.98  3.26  3.57 | 3.42 | 0.09 |
| −Fe + FeSO_4_ | 5.27  6.23  4.22  5.55 | 5.41 | 0.70 | 2.00  2.29  1.72  2.15 | 2.08 | 0.06 | 7.27  8.52  5.94  7.70 | 7.49 | 1.16 |
| −Fe + Fe(II)-EDTA | 5.22  5.05  5.71  6.26 | 5.47 | 0.29 | 2.08  1.91  1.98  2.35 | 2.03 | 0.04 | 7.30  6.96  7.69  8.61 | 7.50 | 0.51 |
| −Fe + Fe(II)-F1 | 7.30  9.40  9.31  10.52 | 9.36 | 1.80 | 2.78  3.50  3.47  3.82 | 3.49 | 0.19 | 10.08  12.80  12.78  14.34 | 12.79 | 3.14 |
| −Fe + Fe(II)-F10 | 6.11  7.38  5.30  7.10 | 6.61 | 0.91 | 2.24  3.06  2.05  2.83 | 2.54 | 0.23 | 8.35  10.44  7.35  9.93 | 9.14 | 2.03 |

**Table 4 Appendix. Biometric data in cucumber grown hydroponically in a nutrient solution**

| Treatments | Dry biomass (mg plant^-1^) | | | | | | | | | | | |
| --- | --- | --- | --- | --- | --- | --- | --- | --- | --- | --- | --- | --- |
|  | Leaves | | | Stem | | | Shoot | | | Root | | |
|  | data points | median | variance measures | data points | median | variance measures | data points | median | variance measures | mean | median | variance measures |
| +Fe | 641  668  646  604 | 644 | 706 | 240  297  259  255 | 257 | 588 | 881  965  905  859 | 893 | 2089 | 184  177  195  187 | 186 | 56 |
| −Fe | 198  231  231  243 | 231 | 374 | 91  104  107  116 | 106 | 107 | 289  335  339  358 | 337 | 857 | 50  67  64  69 | 66 | 74 |
| −Fe + F1 | 235  200  249  245 | 240 | 497 | 117  99  119  121 | 118 | 103 | 352  300  368  366 | 359 | 1012 | 66  59  81  70 | 68 | 85 |
| −Fe + F10 | 209  231  249  250 | 240 | 371 | 98  109  119  109 | 109 | 74 | 307  340  368  359 | 350 | 728 | 59  65  74  68 | 67 | 39 |
| −Fe + FeSO_4_ | 306  266  297  261 | 282 | 499 | 148  125  117  135 | 130 | 179 | 454  391  414  397 | 406 | 806 | 97  75  101  80 | 89 | 161 |
| −Fe + Fe(II)-EDTA | 302  292  318  302 | 302 | 116 | 126  124  144  143 | 135 | 115 | 429  416  461  445 | 437 | 381 | 97  86  101  98 | 98 | 43 |
| −Fe + Fe(II)-F1 | 290  296  310  281 | 293 | 148 | 136  129  140  137 | 137 | 22 | 426  425  450  418 | 426 | 195 | 72  81  86  94 | 84 | 85 |
| −Fe + Fe(II)-F10 | 311  324  304  310 | 311 | 71 | 133  141  145  144 | 143 | 30 | 444  465  449  454 | 452 | 81 | 100  94  87  95 | 95 | 29 |

**Table 5 Appendix.** Active Fe concentration and content in the second leaves (L2) of cucumber grown hydroponically in a nutrient solution

| Treatments | Fe concentration (μg g^-1^) | | | Fe content (μg leaf^-1^) | | |
| --- | --- | --- | --- | --- | --- | --- |
|  | data points | median | variance measures | data points | median | variance measures |
| +Fe | 101  90  66  93 | 91.5 | 227 | 12  11.1  11.4  13.8 | 11.7 | 1.46 |
| −Fe | 21  13  22  21 | 21.0 | 17.6 | 1.0  0.9  1.2  1.4 | 1.10 | 0.05 |
| −Fe + F1 | 15  26  21  19 | 20.0 | 20.9 | 0.9  1.3  1.4  1 | 1.15 | 0.06 |
| −Fe + F10 | 23  21  20  16 | 20.5 | 8.6 | 1.4  1.1  1.3  1.2 | 1.25 | 0.02 |
| −Fe + FeSO_4_ | 65  70  76  72 | 70.0 | 20.9 | 4.0  4.6  4.6  4.4 | 4.5 | 0.08 |
| −Fe + Fe(II)-EDTA | 47  49  54  40 | 48 | 33.7 | 3.8  4.4  3.5  4.1 | 3.95 | 0.15 |
| −Fe + Fe(II)-F1 | 96  76  96  79 | 87.5 | 115.6 | 7.9  7.5  6.6  7.3 | 7.40 | 0.29 |
| −Fe + Fe(II)-F10 | 90  74  101  93 | 91.5 | 128.3 | 6.1  5.4  7.6  6.4 | 6.25 | 0.84 |

**Table 6 Appendix.** Root ferric chelate reductase (FC-R) activity of cucumber grown hydroponically in a nutrient solution

| Treatments | Root FC-R (μmol Fe^2+^ g^-1^ DW h^-1^) | | |
| --- | --- | --- | --- |
|  | data points | median | variance measures |
| +Fe | 3.74  3.11  3.82  3.02 | 3.43 | 0.17 |
| −Fe | 14.00  15.64  9.25  15.69 | 14.82 | 9.20 |
| −Fe + F1 | 17.10  13.31  15.18  13.38 | 14.28 | 3.22 |
| −Fe + F10 | 11.70  14.90  13.65  10.84 | 12.68 | 3.39 |
| −Fe + FeSO_4_ | 5.28  6.51  6.21  5.95 | 6.08 | 0.27 |
| −Fe + Fe(II)-EDTA | 2.83  4.76  2.79  5.57 | 3.80 | 1.96 |
| −Fe + Fe(II)-F1 | 3.63  3.50  1.73  5.77 | 3.57 | 2.73 |
| −Fe + Fe(II)-F10 | 4.23  3.52  5.93  6.10 | 5.08 | 1.62 |
